# Our Relations with Our American Confreres Living and Practicing Abroad

**Published:** 1899-03

**Authors:** 


AMERICAN JOURNAL

—OF—

DENTAL SCIENCE.

Vol. xxxii. Baltimore, March, 1899. No. 11.

### Selections.

ARTICLE I.

### OUR RELATIONS WITH OUR AMERICAN CONFRERES LIVING AND PRACTICING ABROAD.

Report of the Committee on Foreign Relations of the National Association of Dental Faculties.

American dentists practicing in Europe have bitterly complained of the granting of the peculiar American degree to those who are, in foreign countries, considered unqualified. There is no questioning the fact that this has been done in the past. The time once was when foreigners flocked to our shores to complete their dental education by an American course of study, and by the securing of an American degree. This was materially checked by irresponsible institutions which conferred their honors too indiscriminately. The American degree was fast falling into such disrepute that it became necessary to do something, and accordingly this national organization of teachers was formed. I need not enlarge upon its great accomplishments. But unfortunately it was not conceived soon enough. Cause for reproach has already been given, and Europe has not hesitated to take advantage of it to her own benefit and in her own interests, and hence the D. D. S., does not now receive the consideration to which of right it is entitled, nor has sufficient credit been accorded to the work of this association. It takes a long time to live down the bad reputation that may be gained in a day.

Two things are charged by American dentists practicing in Europe:

First that students from the old countries are received by our schools and given advanced standing on the presentation of certificates in foreign tongues which are really worthy no consideration.

Second, that diplomas are practically sold by American institutions, and degrees conferred *in absentia.*

There is, unfortunately, no disputing the fact that our confreres abroad have sometimes had cause for complaint that the value of their diplomas has been depreciated, and that they have not been sufficiently protected by the schools granting them. Ever since the organization of this association of colleges, its rules governing the admission of students have been violated on different occasions, through ignorance of the value of some of the certificates which the regulations have made necessary for advanced standing. It is also more than probable that worthless, and even fraudulent certificates, have sometimes been used as pretexts for giving advanced standing in certain American colleges, when their real character should have been well known by the authorities. A foreigner who desires an American degree, and who occupies but a low social position at home, procures a certificate from some unqualified source, perhaps under false representations. It is written in a foreign tongue and sealed with some pretentious seal, possibly that of an emigration or other bureau. This he presents to the American college, assuring the authorities that it represents a definite course of dental study. The dean is perhaps unable or indisposed to have it verified, and it is accepted, the applicant under it is admitted to the senior course and graduated at the end of a single term. Thus, after an absence of but a few months, the student, perhaps a servant or a barber's apprentice who had become possessed of a little money, returns to his native land and flourishes in the faces of his former associates a diploma that should be the distinguishing characteristic of an educated man, and claims to be the confrere of those who have honestly earned a certificate of fitness from an American school, and upon whose diploma this unmerited scandal and disgrace has thus been brought.

It is possible that the institution thus offending may have been nothing more than careless. It would take weeks to verify the certificate presented, and then it would be too late for entrance. There are no means at hand by which the value of the document can be ascertained, and so the applicant is given the benefit of the doubt and admitted to advanced standing.

Formal complaint was last year made by American dentists in Switzerland in the case of a man named Stauber, who was admitted to the senior class of a college having membership in this body. He had been permitted to join upon the presentation of a foreign certificate. Culpable negligence seemed to have been exercised, and had it not been for the energetic protest of our confreres abroad the student would have been graduated at the end of a few months. Upon the presentation of the case the college reduced Stauber to the Freshman class, and he must wait for his diploma. It is further charged that he was matriculated when not in this country, the date of the closing of time for registration having expired before his arrival in America. Such cases as this should be closely investigated, that the offending college may be punished if guilty, or exonerated if innocent.

It has appeared impossible in many instances to determine the character of the certificates presented. The foreign school, or pretended school, is unknown here.

We have no list of such, and great injustice might be done to applicants if the document is refused, provided it be genuine and sufficient. But it is quite proper for every college to insist upon the endorsement of some known authority. If, as it now is, the authorities are conscientious and ask for a verification of the document presented, the prospective student perhaps brings a countryman who is suborned to give a false interpretation of it. Or he goes to a rival school, representing that the first to which he applied, and which calls for the additional testimony, had accepted him, but that he had found the college to be inferior to its neighbor, and so he wishes to transfer his matriculation to a better one. That appeals to more than one perverted sense, and he is accepted on his mere assertion, skillfully made, that the document had been approved by the other institution, and is given advanced standing.

As for the determination of preliminary qualifications, that is a yet more difficult affair. The systems of general education in different countries are so diverse that it is almost an impossibility to decide what may be accepted as the equivalent for the standard of the National Association of Dental Faculties. And so the reception of students from abroad is that which the most conscientious dean may be at fault.

This condition of affairs has long existed. It forms the basis for many bitter complaints on the part of both foreign and American dentists practicing abroad. It very loudly calls for reform. The good name and reputation of this association is concerned, and we are in honor bound to seek some remedy.

The communications from abroad that have been referred to this committee suggest that a board of European dentists should be appointed by this body, who shall take cognizance of such cases, and whose endorsement of the status of a proposed student shall be necessary for his matriculation in any recognized college. To give to such a foreign and irresponsible board plenary powers in the acceptance of applicants for matriculation from abroad is, of course, quite impossible. We have no legal or moral right to delegate the authority that has been by law vested in the responsible faculties of our colleges. In some of the States the determination of the qualification is vested in State authorities, and they could not and would not delegate it to any board whatever. In the State of New York the college officers have nothing to do with the determination of the preliminary qualifications of applicants. They must obtain from the State regents a dental student's certificate before they can be accepted.

But your committee can see no objection to the naming of an advisory board, whose endorsement of any paper and whose certificate of educational and moral status may be considered sufficient, and it therefore recommends that three qualified persons, resident in each of the principal countries of Europe, be appointed as an advisory board, to whom students from abroad may present their certificates of qualification and moral character for endorsement, or ot whom the papers of students from abroad concerning which there is doubt or uncertainty, may be referred for authentication and approval. Your committee was advised that this matter would be brought before the American Dental Society of Europe. at its meeting in London, August 1st, and that the chairman would be apprised of any action there taken.*

---

*Since this report was presented and adopted the chairman of the committee has received an abstract of the proceedings, in which was recommended the very action taken by the National Association of Dental Faculties at its late annual meeting.

